# The mitochondrial derived peptide humanin is a regulator of lifespan and healthspan

**DOI:** 10.18632/aging.103534

**Published:** 2020-06-23

**Authors:** Kelvin Yen, Hemal H. Mehta, Su-Jeong Kim, YanHe Lue, James Hoang, Noel Guerrero, Jenna Port, Qiuli Bi, Gerardo Navarrete, Sebastian Brandhorst, Kaitlyn Noel Lewis, Junxiang Wan, Ronald Swerdloff, Julie A. Mattison, Rochelle Buffenstein, Carrie V. Breton, Christina Wang, Valter Longo, Gil Atzmon, Douglas Wallace, Nir Barzilai, Pinchas Cohen

**Affiliations:** 1 Leonard Davis School of Gerontology, University of Southern California, Los Angeles, CA 90089, USA; 2 Department of Medicine, The Lundquist Institute at Harbor-UCLA Medical Center, Torrance, CA 90502, USA; 3 Department of Physiology, The Barshop Institute, University of Texas Health at San Antonio, TX 78229, USA; 4 Translational Gerontology Branch, National Institute on Aging, Dickerson, MD 20892, USA; 5 Calico Life Sciences, South San Francisco, CA 94080, USA; 6 Department of Preventive Medicine, Keck School of Medicine, USC, Los Angeles, CA 90089, USA; 7 Department of Medicine, Albert Einstein College of Medicine, Bronx, NY 10461, USA; 8 Department of Human Biology, Faculty of Natural Science, University of Haifa, Haifa, Israel; 9 Center for Mitochondrial and Epigenomic Medicine, Children’s Hospital of Philadelphia, Department of Pediatrics, Division of Human Genetics, Perelman School of Medicine, University of Pennsylvania, Philadelphia, PA 19104, USA

**Keywords:** aging, mitochondria, peptides, humanin

## Abstract

Humanin is a member of a new family of peptides that are encoded by short open reading frames within the mitochondrial genome. It is conserved in animals and is both neuroprotective and cytoprotective. Here we report that in *C. elegans* the overexpression of humanin is sufficient to increase lifespan, dependent on *daf-16/Foxo*. Humanin transgenic mice have many phenotypes that overlap with the worm phenotypes and, similar to exogenous humanin treatment, have increased protection against toxic insults. Treating middle-aged mice twice weekly with the potent humanin analogue HNG, humanin improves metabolic healthspan parameters and reduces inflammatory markers. In multiple species, humanin levels generally decline with age, but here we show that levels are surprisingly stable in the naked mole-rat, a model of negligible senescence. Furthermore, in children of centenarians, who are more likely to become centenarians themselves, circulating humanin levels are much greater than age-matched control subjects. Further linking humanin to healthspan, we observe that humanin levels are decreased in human diseases such as Alzheimer’s disease and MELAS (Mitochondrial Encephalopathy, Lactic Acidosis, and Stroke-like episodes). Together, these studies are the first to demonstrate that humanin is linked to improved healthspan and increased lifespan.

## INTRODUCTION

Mitochondria are central to several theories of aging as they are the major producer of both energy and free radicals, they regulate cell apoptosis, and their dysfunction is central to the observed physiological declines that occur during the aging process [1–5]. Similarly, mitochondrial dysfunction is found in many age-related diseases, although whether this is causal or simply correlative has yet to be established [6–9]. Furthermore, in addition to their role in energy utilization and cell survival, alterations in mitochondrial genes can increase lifespan in several model organisms and mitohormesis has been implicated in lifespan [10–16]. Interestingly, although the vast majority of the proteins found within the mitochondria are encoded by the nuclear genome, mitochondria have their own unique translational machinery and genome, which was previously believed to only code for 13 proteins [17, 18].

In the past decade the number of identified mitochondrial derived peptides (MDPs) and micropeptides have grown exponentially [19, 20]. Micropeptides are an emerging group of small (<100 amino acids) proteins that are often encoded within long-noncoding RNAs, introns, or 5’ and 3’ untranslated regions [21–26]. Perhaps because of the compact nature of the mitochondria and absence of introns, MDPs are encoded in alternative open reading frames within known genes. These MDPs are a novel group of micropeptides encoded within the mitochondrial genome and have been shown to have a large number of biological effects [27–32].

Humanin is the first member of this new class of mitochondrial-derived signaling peptides that now includes MOTS-c and SHLP1-6 [19, 20]. The humanin gene is found as a small open reading frame within the 16s rRNA gene of the mitochondrial genome. It is highly conserved in chordates but can also be found in species as distant as the nematode [33], suggesting that humanin is an ancient mitochondrial signal used to communicate to the rest of the organism. It was initially discovered in a screen for proteins that protected against Alzheimer's disease (AD), but was also independently found to bind IGFBP3 and Bax [33–35]. Since its initial discovery, humanin’s role in protecting against many other age-related diseases such as atherosclerosis and stroke has expanded with numerous beneficial effects now evident [36–38]. Circulating levels of humanin correlate with lifespan in different mouse models of aging and bi-weekly injections of the humanin analogue HN-S14G delays the cognitive decline in mice [39–42]. Another MDP, MOTS-c, has already been associated with longevity in centenarians [43], although association studies of humanin levels in humans have been equivocal [44–46]. In this study we further investigated the relationship between humanin, longevity, and healthspan using transgenic worms, multiple mammalian species with divergent lifespans, and also the children of centenarians compared to age-matched controls.

## RESULTS

### Humanin overexpression is sufficient to increase lifespan in worms

**Figure 1:**
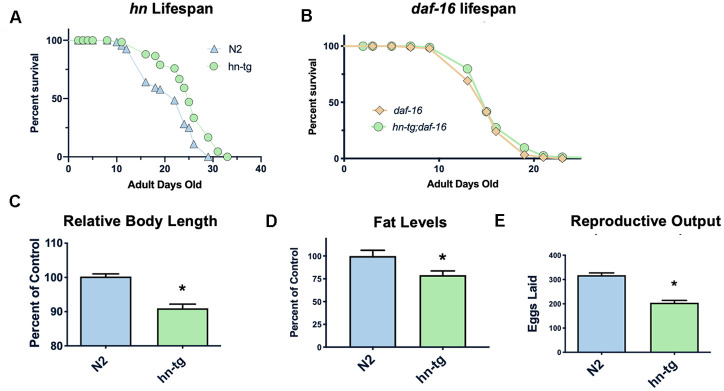
**Humanin overexpression is sufficient to increase lifespan in *C. elegans*.** HN overexpression significantly increased lifespan in worms (average lifespan of 19.0 days) compared to wild-type/N2 (average lifespan 17.7 days) **(A).** This increase in lifespan was dependent on *daf-16* as *daf-16(mu86)* mutants did not have any increase in lifespan when crossed with the humanin-tg strain (average lifespan 15.5 days vs 16.1 days respectively) (p < 0.2) (**B**). Hn-tg worms also had a significant decrease in body length, body fat, and reproductive output (**C**–**E**). *indicates p<.05.

Humanin has been shown to protect against many toxic insults. For example, we found that humanin treatment protected against lethal doses of heat shock in yeast (Supplementary Figure 1). To test if humanin is sufficient for an increase in lifespan, we generated a transgenic worm overexpressing humanin using a ubiquitous promoter (*ife-1*) and MoscI technology (Knudra, Murray, UT). After backcrossing the worms to wild-type/N2 worms six times, we examined their lifespan and found that the transgenic worms had a small but consistent and significant (p<.05) increase in lifespan compared to wildtype/N2, indicating that humanin is sufficient to increase lifespan in worms (Figure 1A). Using epistatic analysis, we found that the increase in lifespan was dependent on the *daf-16*/FOXO gene as humanin overexpression does not increase lifespan in a *daf-16(mu86)* deficient strain (Figure 1B). Humanin's interaction with the insulin/IGF signaling pathway has been previously shown in mice, suggesting that this is a conserved mechanism of action for humanin [39, 47]. Phenotypic analysis of these transgenic worms also found them to have a decrease in body size, body fat, and reproductive output (Figure 1C–1E).

### Humanin mice are protected from toxic insult and phenocopy the transgenic worms

**Figure 2:**
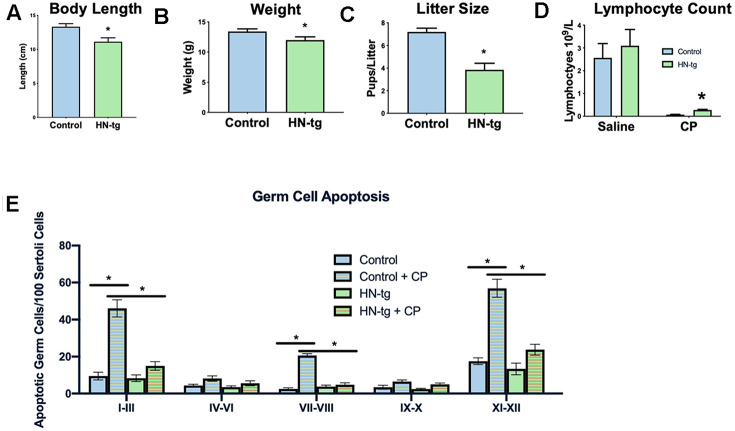
**HN-tg mice phenocopy the transgenic worms and are protected from toxic insult.** Humanin transgenic mice have a significant decrease in body length by 12% at 28 days of age (n= 5 for control and n=3 for the hn-tg mice) (**A**). Body weight at the same age was also decreased by 10.4% (n=26 and n=16 for control and hn-tg mice respectively) (**B**), while litter size decreased by 46.5% (n=10 and n=15 for control and hn-tg mice respectively) (**C**). When administered cyclophosphamide, mice have a decreased lymphocyte count and transgenic mice are protected from this toxin (n=6 or 7 per group) (**D**). Similarly, when examining germ cell apoptosis organized by spermatogenic stages in the same cyclophosphamide treated mice, humanin transgenic mice are significantly protected from CP induced apoptosis (**E**). * indicates p<.05.

Having created transgenic worms, we next developed a transgenic mouse model using a construct that included the humanin-ORF driven by a CMV promoter to test the effects of long-term exposure to humanin. Mice harboring the humanin transgene were both viable and fertile. We have previously published that there is a 16% increase in circulating humanin levels in these mice [48]. With this model, the effects of long-term exposure to humanin were assessed. Phenocopying the worms, the mice had a decrease in body length, body weight, and litter size (Figure 2A–2C). To further assess the HN-tg mice and because humanin has been shown to be cytoprotective by many different labs, we utilized the well-established cyclophosphamide toxicity model. Cyclophosphamide (CP) is a chemotherapeutic agent that causes many side effects, but humanin administration has been shown to prevent many of these [49]. Similar to previous studies that administered humanin exogenously, humanin transgenic mice were also protected from many of the side effects of CP such as the decrease in lymphocytes and increase in apoptosis in germ cells (Figure 2D, 2E). Under control conditions, there were no differences in germ cell apoptosis between control and HN-tg mice, suggesting that germ cell apoptosis was not the cause of the decreased fertility in these mice (Figure 2E).

### Humanin treatment in middle-aged mice improves metabolic health

**Figure 3:**
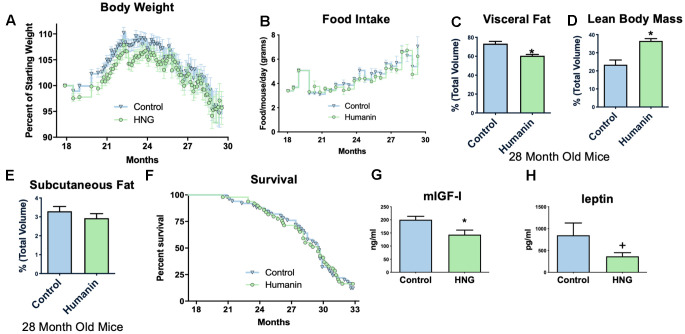
**Midlife humanin treatment improves metabolic health in mice.** Twice weekly treatment with HNG in midlife improves weight (**A**) without changing food intake (**B**). There were also improvements in body composition with a decreased values in visceral fat (**C**), an increase in lean body mass (**D**), and no change in subcutaneous fat (**E**). Although there was no significant difference in lifespan with this low of a dose of humanin (**F**), there was a significant decrease in circulating IGF-I (**G**) and a trend (p<.1) for leptin (**H**). *indicates p<.05 +indicates p<.10.

To examine if humanin treatment can increase the lifespan and healthspan in a mammalian model, 18-month-old, female C57BL/6N mice were obtained from the NIA and administered bi-weekly HNG (4 mg/kg, IP), a potent humanin analogue. Body weight was significantly reduced compared to control/vehicle injected until older ages (Figure 3A), but food intake did not significantly differ between HNG-treated and control mice during this same time period (Figure 3B), suggesting a possible metabolic effect as opposed to the treatment causing illness. At 28-months of age, body composition was determined by micro-CT [50] and even without a significant difference in body weight at this time point, the HNG-treated mice had a decrease in visceral fat (Figure 3C), an increase in lean body mass (Figure 3D), and no change in subcutaneous fat (Figure 3E) (N=5/group). After 14-months of treatment, there was no significant difference in lifespans between the groups (Figure 3F). However, the HNG-treated group showed improvements in healthspan related parameters such as a significant decrease in IGF-I and trend for a decrease in leptin (Figure 3G, 3H).

### Humanin levels are inversely associated with disease and positively associated with lifespan

**Figure 4:**
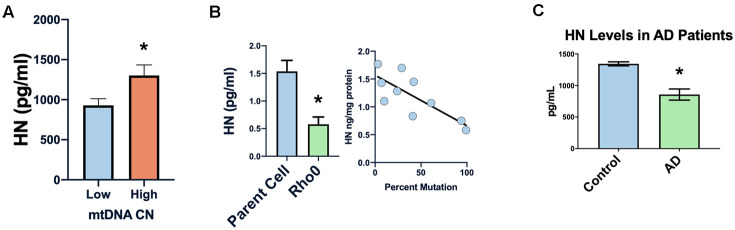
**Humanin levels are related to human mitochondrial health.** Low mtDNA copy number is associated with lower levels of humanin (**A**). Increased mtDNA mutation or absence of mtDNA (rho0 cells) is associated with decreased levels of humanin (**B**). Humanin levels are also decreased in the CSF of Alzheimer’s diseases patients (n=4) compared to control (n=3) (**C**) *indicates p<.05.

In many diseases the mitochondrial DNA copy number decreases, and we found a correlation between peripheral blood mononuclear cells’ mtDNA copy number and humanin levels in newborn cord blood (Figure 4A) [51–53]. Furthermore, in cell lines with the 3243 MELAS (mitochondrial myopathy, encephalopathy, lactic acidosis, and stroke-like episodes syndrome) mutation, humanin levels were inversely correlated with the percent of mutated mitochondria (Figure 4B), suggesting that humanin could be a marker of the mitochondrial dysfunction that occurs with increasing age. Humanin levels in the cerebral spinal fluid of patients with AD (n=3) was significantly lower compared to controls (n=4) (Figure 4C).

**Figure 5:**
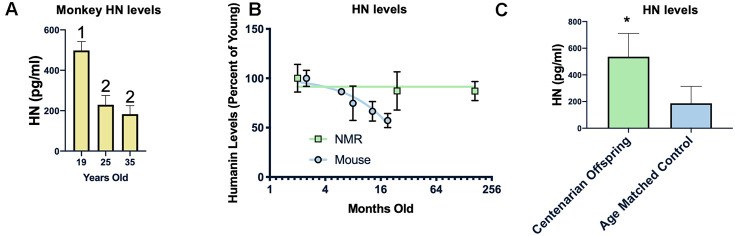
**Humanin levels are correlated with longevity.** HN levels decline with age in monkeys between 19 and 25 years of age but not between 25 and 35 years of age (n=40, 25, and 21 for 19, 25, and 35 years of age respectively) (**A**). In two rodents, the short-lived mouse (n=29) has a decline in humanin levels over the first 16 months of life, while the long-lived naked mole rat (NMR) (n=10) maintains levels over 2 decades (**B**). Humanin levels in offspring of centenarians (n=18), who have a higher chance of becoming centenarians themselves, are significantly higher than age-matched control levels (n=19)(**C**). * indicates p<.05, columns with different numbers are significantly different from each other.

Examining the circulating humanin levels in a number of species we found a decrease with age in rhesus macaques, and a dramatic decrease between 19 and 25 years of age (Figure 5A). As previously reported, we also found a decrease in humanin levels in mice (Figure 5B), with an approximately 40% decrease over the first 18 months of life. We also assessed the circulating levels of humanin in the longest-lived rodent, the naked mole-rat (*Heterocephalus glaber*). In keeping with their negligible senescence phenotype and unchanged risk of dying, there was only a trend towards a decrease in humanin levels over the lifespan of the naked mole-rat (Figure 5B, p=0.08) [54–57]. The naked mole-rat homologue of humanin is 75% identical to the human version and, given this difference, we are only confident in the age-related changes in relative levels of humanin in this species due to possible differences in antibody affinity to NMR humanin. Nevertheless, basal levels of humanin are 4-fold higher than those observed in young mice.

Because of this inverse correlation between age and humanin levels, we examined the circulating levels of humanin in a human centenarian cohort. Given the lack of a control group for centenarians, we examined the children of centenarians who have a decreased amount of disease compared to age-matched controls as well as a decrease in mortality rate and have a propensity to live longer [58, 59]. As shown in Figure 5C, children of centenarians have significantly higher levels of circulating humanin than their control counterparts (N=18 and 19 respectively), further supporting a correlation between humanin levels, diseases of aging, and lifespan.

## DISCUSSION

Humanin is the most conserved MDP and may be an ancient mitochondrial signal used to communicate with the rest of the body. Thus, we took a multi-species approach to examine the relationship between humanin, lifespan, and healthspan. Although previous studies have suggested that humanin could be a novel anti-aging peptide, this study is the first to show that humanin overexpression is indeed sufficient to extend lifespan. We further find that humanin interacts with the insulin/IGF-I pathway to increase lifespan, which is supported by our previous study showing that humanin both affects and is affected by the insulin/IGF pathway in mice [39]. Both transgenic worms and mice were phenotypically similar, and they had a decrease in body size, body weight or fat, and brood size. Interestingly, this same phenotype has been reported in mice with reduced IGF-I levels [60]. A difference in body weight and fat was also observed in our studies in middle-aged mice that were lighter and had less visceral fat when treated with HNG compared to control. Similarly, in human centenarians, we have previously reported a decrease in the number of children compared to control, further supporting the link between high humanin levels and a decrease in reproduction [61]. We and others have also found decreased levels of circulating IGF-I in the offspring of centenarians, further supporting this link [62, 63]. This decrease in reproduction is a common thread throughout a number of different longevity models and is likely due to the natural tradeoff of reproduction for longevity [2, 64–66].

Having established that humanin is sufficient to increase lifespan in worms, we examined if humanin treatment started in mid-life would be able to increase lifespan in mice. Administration of HNG to these female mice did not increase lifespan, but it was able to improve metabolic parameters without changing food intake. One possible reason for this lack of lifespan effect could be due to a sub-optimal dosage for lifespan extension as humanin has a half-life of 30 minutes. Alternatively, it may be that the treatment was started too late as it has been previously shown that the lifespan extending effects of IGF-I inhibition is required during early life. In that publication, late life inhibition only lead to an increase in healthspan, similar to what we saw in our study [67]. In previous publications we found that cognitive decline, inflammatory markers such as IL-6 and Iba-1, rotarod performance, and fibrosis was improved in this cohort of mice, suggesting that this dosage is sufficient to improve specific aging phenotypes [41, 68]. In this study, midlife humanin treatment attenuated the obesity that occurs in mid-life, but did not exacerbate the gradual decline in bodyweight at older ages. This improvement in metabolic parameters has been previously seen in mice treated with HNG and in fact, human studies have found that HN levels were negatively correlated with HbA1c levels and triglyceride levels [29, 44]. Taken together, even at the low doses that we administered to middle-aged mice, humanin treatment improved several lifespan and healthspan markers such as metabolism, fibrosis, and cognitive decline [41, 68].

Humanin-tg mice were protected from cyclophosphamide treatment to the same extent as our previous study showing that C57BL6/J mice injected with humanin were also protected from cyclophosphamide [49]. Combined with our previous publication showing that the humanin-transgenic mice were equally protected from glucocorticoid toxicity as humanin injected mice [48], these results demonstrate that our humanin-transgenic model is phenotypically equivalent to daily injections of humanin and in future studies we will examine the lifespan of the humanin transgenic mice. Based on these results combined with those in worms, we predict a lifespan increase in these mice.

Moving from model organisms to humans, we found that a decrease in humanin levels correlates with a number of disease parameters. Humanin levels are inversely correlated with a decrease in mitochondrial DNA copy number, which in itself has been associated with a number of different diseases such as cancer, kidney disease, and cardiovascular disease [51–53]. Although we do not believe that circulating humanin levels derive from PBMCs, we think that this change in PBMC DNA copy number is a surrogate marker for other diseases. Humanin levels are also inversely correlated with the heteroplasmy levels in MELAS cells and it is well recognized that heteroplasmy levels are associated with disease severity. Many studies have shown that humanin can protect animals in several models of AD and we find that AD patients have a decrease in CSF humanin. Thus, from many different models of human disease we find a correlation between low humanin levels and disease that is compatible with the hypothesis that humanin is a regulator of lifespan and healthspan.

Over a lifespan, humanin levels have been shown to decrease and in rhesus monkeys and mice, this trend holds true. Due to the relatively-high interspecies sequence similarity of humanin (92%, 73%, 75% in monkeys, mice, and NMR respectively) (Supplementary Figure 2), we were able to measure humanin levels in other species, although the absolute levels may not be completely accurate due to differences in antibody affinity to the species-specific humanin. Our results regarding the humanin levels of naked-mole rats is particularly intriguing as this extraordinarily long-lived (>30y), mouse-size rodent shows no age-associated exponential increase in risk of dying [57], nor is there evidence of the decline in physiology that manifests as mammals get older [54, 69, 70]. For example, even into the third decade of life, there are no significant changes in cardiac function, body composition, reproductive capacity, and metabolism [71, 72]. Part of this remarkable physiology may be because mitochondrial function is protected in this species. Hints of this are evident from the peroxidation-resistant phospholipid composition of mitochondrial membranes [73]. Moreover, humanin may play an integral cytoprotective role, protecting against the vagaries of oxidative stress and thereby facilitating tissue homeostasis. Given the many documented cytoprotective effects of humanin in chronic age-associated diseases, sustained high levels of humanin likely play a pivotal role in the observed negligible senescence observed in naked mole-rats.

Similar to the naked mole rat, centenarians and their children are remarkably healthy. Children of centenarians also have higher humanin levels compared to control and this may reflect a higher baseline level of humanin at younger age or a decrease in rate of humanin decline. Our data from newborn cord blood, mitochondrial copy number, and humanin levels demonstrate that differences in humanin levels and mitochondrial copy number may exist starting from birth, further supporting the idea that the elevated levels of humanin in centenarians may also start from birth. Either way, the centenarian data further links longevity, health, and humanin levels.

Mitochondria play a large role in aging and age-related diseases and mitochondrial signals have been shown to increase lifespan. Here we have found that humanin, and perhaps other mitochondrial derived peptides, is not only correlated with health and lifespan but can significantly improve both parameters on its own.

## MATERIALS AND METHODS

### *C. elegans* fat measurement

Fat staining and quantification was performed as previously described, but with the modification of the use of neutral buffered formalin (NBF) instead of MRWB buffer used for fixation [74, 75]. In short, 1-day old adult worms were fixed with NBF, and then freeze-thawed three times to help permeabilize the cuticle and cells. Nile red diluted in water at a final concentration of 100ng/mL was used to stain the worms overnight. Quantification was performed using ImageJ.

### *C. elegans* lifespan studies

Worms were synchronized by hypochlorite treatment and allowed to grow to young adults at 15°C. Approximately 100 worms per group were then transferred to fresh NGM plates containing 10μM FuDR and they were subsequently maintained at 20°C. Worms were scored as alive or dead approximately every other day and transferred to fresh plates once a week. Worms that had internal bagging or ruptured vulvas were censored from the study. Each lifespan study was also repeated a minimum of 3 times. The experiments were also performed without FuDR with similar results. *daf-16(mu86)* worms were used for these experiments.

### *C. elegans* body length measurements

Worms were synchronized by hypochlorite treatment and allowed to grow to young adults at 20°C. Pictures were taken on adult day 1 or 2 and length was measured using the ImageJ software.

### *C. elegans* brood size

Worms were synchronized via hypochlorite treatment and L1 worms were individually placed on separate plates. Worms were transferred to a fresh plate every day and the total number of eggs and larva were counted.

### *C. elegans* transgenic generation

The humanin transgenic worm was created by Knudra Transgenics (now known as NemaMetrix Inc). Transgenic worms were created using *MoscI* technology to insert a single copy of the humanin open reading frame into the genome of the worm and this was verified by PCR [76]. The *ife-2* promoter was used as well as the *tbb-2* (UTR) to flank the humanin gene.

### Humanin mouse lifespan study

18-month old, female mice were obtained from the NIA aged mouse colony (N=100) and were given IP injections with either the humanin analogue S14G or water/vehicle twice a week at 4mg/kg for 14 months [41]. Survival was monitored daily and both weight and food intake were measured weekly. All experiments were approved by the University of Southern California’s Institutional Animal Care and Use Committee under protocol #20787.

### Mesoscale discovery measurements

Measurement of leptin and IGF-I were obtained according to the manufacturer’s instructions.

### Humanin ELISA

Humanin levels of plasma were measured by our in-house HN ELISA developed as previously described [47]. Briefly, plasma HN was extracted in 90% acetonitirile and 10% 1N HCl in a 2:1 ratio of extraction buffer to plasma. The mixture was incubated at room temperature for 30 minutes and then dried by speedvac. 200μL of phosphate buffer was added to the dried extract and then used for the assay that employs a polyclonal, rabbit antibody. Absorbance was measured on a plate spectrophotometer (Molecular Designs, Sunnyvale, CA, USA) at 490 nm.

### Humanin transgenic-mice

The creation of this strain has been previously described [48]. In brief we developed a transgenic mouse model using a construct that included the humanin-ORF driven by a CMV promoter that was injected into the pronucleus of fertilized B6D2F1 mice ova. To obtain a congenic line, the transgenic mice were backcrossed into the C57BL/6 strain. Transgenic mice were genotyped using mouse tail DNA via PCR.

### Naked mole rats

Naked mole-rat colonies were maintained at the University of Texas Health Science Center vivarium under previously described conditions [57]. Animals were euthanized via isoflurane followed by cardiac exsanguination. Flash-frozen liver samples from both male and female mole-rats ranging in age from 1 year to 21 years were used in this study. The NMR homologue of humanin is 75% identical to the human version (Supplementary Figure 2). Given this difference, we are only confident in the relative levels of humanin in this species due to possible differences in antibody affinity to NMR humanin.

### NIA monkey study

All procedures were approved by the NIA Intramural Research Program Institutional Animal Care and Use Committee. Rhesus monkeys (*Macaca mulatta*) were control subjects from an ongoing longitudinal study of calorie restriction and longevity. Monkeys were housed at the NIH Animal Center in standard primate caging with controlled temperature and humidity and a 12-hour light cycle. They were fed a natural ingredient diet containing 56.9% carbohydrate, 17.3% protein and 5% fat at approximately ad libitum levels. Water was available ad libitum. Samples were collected following an overnight fast under anesthetized conditions.

### Body fat measurements

Body fat was measured as described previously [50]. Briefly, at 28 months of age, mice (representing average body weight, N=5/group) the abdominal region was scanned with a Siemens InveonCT scanner. Two-dimensional gray-scale image slices were reconstructed into a three-dimensional tomography using COBRA software.

### Apoptosis and cyclophosphamide experiments

We generated HN transgenic (HN-tg) mice expressing a CMV-promoter driven humanin transgene as previously described [48]. After genotyping, 1) groups of 7 adult (5-10 month-old) HN-tg and age-matched wildtype (WT) mice were used for the characterization of male reproductive phenotype, and 2) groups of 6 adult HN-tg and age-matched WT mice were treated with a single-dose of CP injection (i.p. 200mg/kg) to examine male germ cell apoptosis at 2 days after treatment. Body and testis weights were recorded at autopsy. Blood samples were collected from the right ventricle of each mouse immediately after death and used for complete blood count using an automated cell counter (VetScanHM2; ABAXIS). Apoptotic germ cells were detected by TUNEL assay as described earlier [49]. The rate of germ cell apoptosis was quantified from 6 mice per groups and expressed as apoptotic index (AI), which is the number of apoptotic germ cells/100 Sertoli cells on testicular sections as previously described [77].

### Centenarian studies

The Ashkenazi Jews (AJ) cohort in the present study, was recruited as previously described [58, 78–80]. This cohort consists of three groups: centenarians, their offspring, and controls of European decedent. A detailed medical history questionnaire was administered, and a physical examination on the AJ was performed as previously described [58, 78, 80]. Informed written consent was obtained from the participants in accordance with the policy of the Committee on Clinical Investigations of the Albert Einstein College of Medicine.

### mtDNA copy number study in humans

mtDNAcn was measured in 73 newborns from the University of Southern California Maternal and Children Health (MACHS) birth cohort study. Mother-child pairs were recruited on the labor and delivery ward at the Los Angeles County + University of Southern California Medical Center from September 2012 to August 2015. Written informed consent was obtained from each pregnant woman prior to any testing. Exclusion criteria included <18 years of age, HIV positive status, physical, mental, or cognitive disabilities that prevented participation, current incarceration, or multiple pregnancy. A 15-cc cord blood sample for each participant was collected by hospital providers in 1 EDTA tube for plasma and DNA isolation. Relative mitochondrial copy number (mtDNAcn) was measured by qPCR assay by determining the ratio of mitochondrial (Mt) copy number to a single copy gene (human [beta] globin: hbg) number in experimental samples relative to a reference as described in the *in vitro* experiments.

### MELAS and Rho0 cells

MELAS cybrids and Rho0 cells were created as previously published [81, 82]. Levels of humanin were measured from cell lysates.

### Alzheimer’s disease CSF samples

Alzheimer’s disease samples were obtained from the UCLA AD Center from patients between the ages of 60-80 years of age. The average age of the groups was not significantly different, and the control group’s average age was 71.0 ±4.3 (SEM) while the AD samples’ was 76.0 ±2.1 years.

### Data availability

The data that support the findings of this study are openly available in figshare at http://doi.org/10.6084/m9.figshare.11783616

## Supplementary Material

Supplementary Figures
