# Supplementary material for: The mitochondrial derived peptide humanin is a regulator of lifespan and healthspan
Source: Aging (Albany NY). 2020 Jun 23;12(12):11185–99. doi: 10.18632/aging.103534 (PMC7343442; doi:10.18632/aging.103534)
Supplement: Supplementary Figures [file aging-12-103534-s001..pdf]

SUPPLEMENTARY FIGURES

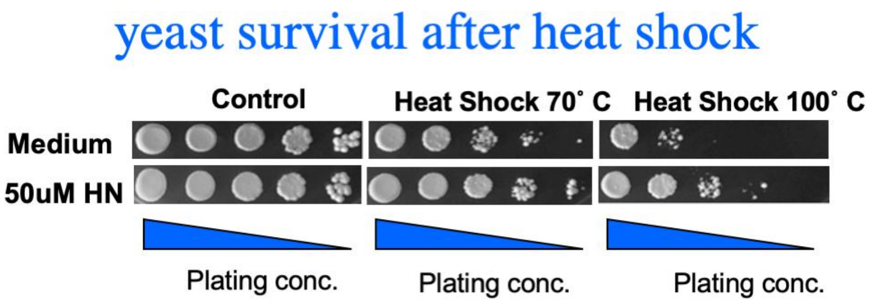

Supplementary Figure 1. HN protects yeast from heat shock. Compared to medium only, yeast treated with 50uM of humanin were protected from both a 70°C and 100°C heat shock.

Human (*H. sapiens*)	MAPRGFSCLLLLTSEIDLPVKRRA	(Cyto)
	MAPRGFSCLLLLTSEMDLPVK	(Mito)
	MAPRGFSCLLLSTSEIDLPVKRRA	(NUMT Chr 3)
	MAPRGFSCLLLSTSEIDLPVKRRA	(NUMT Chr 11)
Rhesus monkey (*M. mulatta*)	KAPRGFNCLLLLTSEIDLPAKRRT	(Cyto)
	KAPRGFNCLLLLTSEIDLPAK	(Mito)
	MAPRGFNCLLLLTSEIDLPVKRRT	(NUMT Chr1)
NMR (*H. glaber*)	MAKRRFFCLLLSVSEIDLPVKRRG	(Mito)
Mouse (*M. musculus*)	TAKRGSNCLLSLISEIDLSVKRLKYNNKTRRPYGA	(Cyto)
	GLAWTAKRGSNCLLSLISEIDLSVK	(Mito)
	MAKGGFNCLLFLISEIDLSVKR	(NUMT Chr6)

Supplementary Figure 2. Humanin is a conserved peptide. Human humanin is highly conserved whether cytoplasmic (Cyto) or mitochondrial (Mito) translation occurs. Furthermore, there are several nuclear to mitochondrial sequences (NumtS) that could possibly generate peptides that would likely be cross reactive with our polyclonal antibody used in the ELISA as well.
